# Prevalence of Cardiovascular Disease and Risk Factors in Ghana: A Systematic Review and Meta-analysis

**DOI:** 10.5334/gh.1307

**Published:** 2024-02-20

**Authors:** Alfred Doku, Lawrence Sena Tuglo, Vincent Boima, Francis Agyekum, Pearl Aovare, Martha Ali Abdulai, Anthony Godi, Ron J. G. Peters, Charles Agyemang

**Affiliations:** 1 Department of Medicine and Therapeutics, University of Ghana Medical School, Accra, Ghana; 2 Department of Public and Occupational Health, University of Amsterdam Medical Centre, University of Amsterdam, Netherlands; 3 National Cardiothoracic Centre, Korle-Bu Teaching Hospital, Accra, Ghana; 4 Department of Nutrition and Dietetics, School of Allied Health Sciences, University of Health and Allied Sciences, Ho, Ghana; 5 Department of Epidemiology, School of Public Health, Nantong University, 9 Seyuan Road, Nantong, Jiangsu, China; 6 Kintampo Health Research Centre, Research and Development Division, Ghana Health Service, P.O Box 200, Kintampo-B/E, Ghana; 7 Department of Biostatistics, School of Public Health, College of Health Sciences, University of Ghana, Accra, Ghana; 8 Department of Cardiology, University Amsterdam Medical Center, University of Amsterdam, Netherlands

**Keywords:** prevalence, cardiovascular disease, risk factors, systematic review, meta-analysis and Ghana

## Abstract

**Background:**

The increasing cardiovascular disease (CVD) burden threatens the global population as the major cause of disability and premature death. Data are scarce on the magnitude of CVD among the population in West Africa, particularly in Ghana. This study examined the available scientific evidence to determine the pooled prevalence (PP) of CVD and risk factors in Ghana.

**Methods:**

We searched electronic databases such as PubMed, Google Scholar, the Cochrane Library, Science Direct and Africa Journal Online databases to identify literature published from the start of the indexing of the database to 10^th^ February 2023. All articles published in the English language that assessed the prevalence of CVD or reported on CVD in Ghana were included. Two authors independently performed the study selection, assessed the risk of bias, extracted the data and checked by the third author. The effect sizes and pooled odds ratio (POR) were determined using the random-effects DerSimonian-Laird (DL) model.

**Result:**

Sixteen studies with 58912 participants from 1954 to 2022 were included in the meta-analysis. Six studies out of 16 reported more than one prevalence of CVD, giving a total of 59 estimates for PP. The PP of CVD in the general population in Ghana was 10.34% (95% Cl: [8.48, 12.20]; l^2^ 99.54%, *p* < 0.001). Based on the subgroup analysis, the prevalence of CVD was higher in hospital-based settings at 10.74% (95%, confidence interval [Cl]: 8.69, 12.79) than in community-based settings at 5.04% (95% Cl: 2.54, 7.53). The risk factors were male gender (pooled odds ratio [POR]: 1.66; 95% CI: 1.02, 2.70), old age (POR: 1.32; 95% CI: 1.21, 1.45), unemployment (POR: 2.62; 95% CI: 1.33, 5.16), diabetes (POR: 2.79; 95% CI: 1.62, 4.81) and hypertension (POR: 3.41; 95% CI: 1.75, 6.66).

**Conclusion:**

The prevalence of CVD was high in Ghana. Urgent interventions are needed for the prevention and management of the high burden of CVD and its risk factors.

## 1 Introduction

Globally, cardiovascular disease (CVD) and its risk factors are the leading causes of preventable morbidity and untimely mortality [1,2,3,4]. CVD is a group of disorders of the heart and blood vessels [2,5,6]. They include coronary artery disease (CAD), cerebrovascular disease, peripheral arterial disease (PAD), rheumatic heart disease (RHD), deep vein thrombosis (DVT), congenital heart disease (CHD) and pulmonary embolism (PE) [2,5,6,7]. The modifiable risk factors for CVD are smoking tobacco, hypertension, diabetes, hypercholesterolaemia, physical inactivity, overweight/obesity, unhealthy dietary intake and excessive alcohol intake [2,6], while the nonmodifiable risk factors include age, gender, family history and ethnicity [2,6]. Nonetheless, having a risk factor does not guarantee an individual will develop CVDs, but the chance is higher if care is not taken to manage (control the effect) the risk factor [8].

The global prevalence was 4.2% from 1990 to 2015 [9], 5.5% in sub-Saharan Africa (SSA) [10] and 10.1% in the Middle East [4]. According to studies, the total prevalence of CVD is expected to rise rapidly due to population growth and ageing, particularly in Northern Africa, Western Asia, Central and Southern Asia, Latin America and the Caribbean, and Eastern and Southeastern Asia, where the proportion of older people is expected to double between 2019 and 2050 [3,11,12]. CVD-associated deaths occur before the age of 70 years in 80% of low- and middle-income countries (LMICs) [2,6,13].

Even though CVD is largely preventable, it accounts for 32% of all global deaths, of which 85% are due to heart attack and stroke [2,6,13]. Over 70% of CVD deaths occur in LMICs, including those in Africa [14]. It is the leading cause of premature deaths in adults in sub-Saharan Africa (SSA), caused primarily by uncontrolled hypertension [2,5]. However, most countries in SSA do not have the requisite resources or robust health systems to manage CVD [15].

In Ghana, the prevalence of CVD ranges from 0.5% [16] to 65.0% [17]. The data from the National Cardiothoracic Center at Korle-Bu Teaching Hospital (KBTH) showed that 60% of deaths among adults were due to heart-related diseases, with 6.5% and 19% being diabetic and hypertensive patients, respectively [8]. Findings from autopsy reports in KBTH by Wiredu & Nyame [18], Edingion [19], and Sanuade et al. [20], showed that 11.1%, 12.8% and 22.2% of all deaths were due to CVD, respectively.

Reports from KBTH and Komfo Anokye Teaching Hospital (KATH) have indicated that there is a rise in CVD and risk factors such as hypertension, diabetes and obesity, especially among middle-aged Ghanaians [8,21]. Another report showed that heart disease is the leading condition recorded at the accident and emergency centres in the KATH [21]. Based on these reports, paying attention to cardiovascular health and controlling risk factors is essential, and it is time to implement evidence-based strategies and inexpensive policies for the prevention and control of CVD and to monitor outcomes. This review aimed to determine the PP of CVD and risk factors in Ghana to assist health professionals, researchers, and policymakers in designing effective interventions for early prevention and management.

## 2 Methods

We performed a systematic review and meta-analysis of studies reporting the prevalence of CVD in Ghana following the Preferred Reporting Items for Systematic Reviews and Meta-Analyses (PRISMA) guidelines and registered on PROSPERO (registration ID: CRD42023395652).

### 2.1 Search strategy

A literature search was conducted from the start of the indexing of the database to 10^th^ February 2023, using PubMed, Google Scholar, the Cochrane Library, Science Direct and the Africa Journal Online databases. The following Medical Subject Headings (MeSH) terms were used: ‘prevalence’ AND ‘risk factors’ AND ‘cardiovascular diseases’ OR ‘cerebrovascular disorders’ OR ‘heart diseases’ OR ‘heart failure’ OR ‘myocardial ischemia’ OR ‘coronary artery disease’ OR ‘peripheral arterial disease’ OR ‘rheumatic heart disease’ OR ‘deep vein thrombosis’ OR ‘congenital heart disease’ OR ‘pulmonary embolism’ OR ‘stroke’ AND ‘Ghana’ OR ‘Gold Coast’. The search strategy is presented in the Supplementary file, Table 1, on page 1.

### 2.2 Study selection

**Figure 1:**
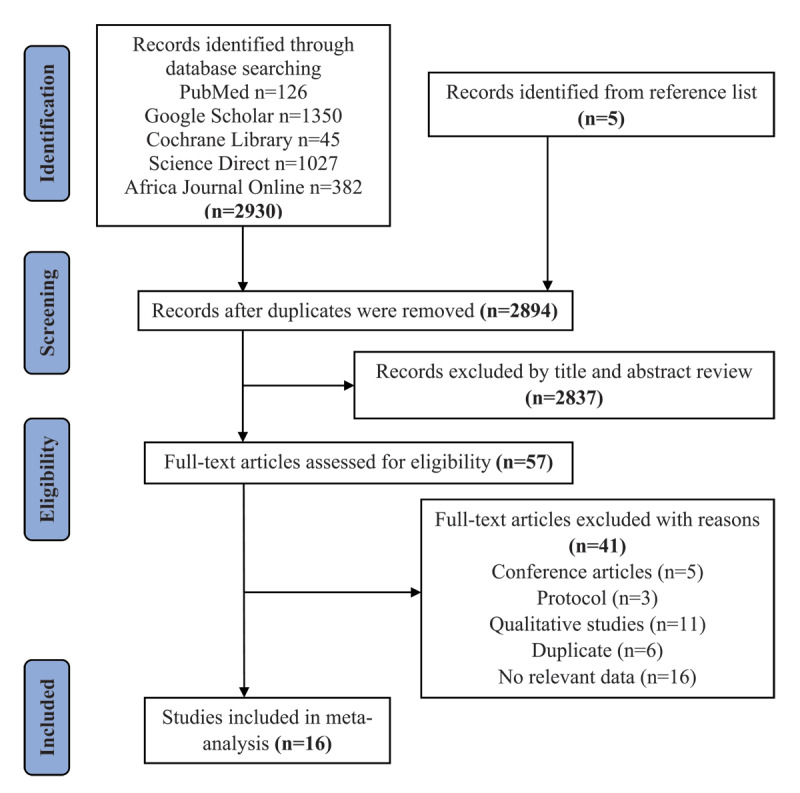
PRISMA flow diagram of study search and selection procedures.

We included articles published in English-language peer-reviewed journals that reported the prevalence of CVD in Ghana (or reported on CVD for which the proportion could be calculated). We excluded opinion papers, qualitative research, comments, conference proceedings, policy papers, letters to the editor, and study protocols without data (Figure 1).

### 2.3 Data extraction

After the database search, duplicates were removed using Mendeley version 1.19.6. Two authors (LST and AD) independently reviewed the search output for eligibility (titles, abstracts then full text) to remove articles that were unrelated to the study question. The full texts of the articles that passed this initial screening were then retrieved and assessed independently by two authors (LST and AD). Any disagreements were resolved by consensus. The data were extracted by two independent authors (LST and AD) using Microsoft Excel. Excel captured the first author or publication year, study population, study setting, study design, regions in Ghana, sample size, CVD diagnosis and reported prevalence (or proportion) of CVD Any disparity in the extracted data by the authors was discussed and resolved by consensus (Table 1).

**Table 1:** Characteristics of the included studies.


STUDY	AUTHOR (PUBLICATION YEAR)	STUDY SETTING	STUDY DESIGN	REGION	DIAGNOSIS (CVD)	SAMPLE SIZE	DISEASED	PROPORTION

1	Sarfo et al., 2018 [24]	Hypertension and Diabetes Clinic	Prospective cohort study	More than one	Stroke	3220	54	1.68

2	Owusu et al., 2018 a [25]	Cardiac Clinic	Cross-sectional study	Ashanti	Hypertensive heart disease	432	154	35.6

	Owusu et al., 2018 b [25]	Cardiac Clinic	Cross-sectional study	Ashanti	Valvular heart disease	432	85	19.7

	Owusu et al., 2018 c [25]	Cardiac Clinic	Cross-sectional study	Ashanti	Cardiomyopathies	432	80	18.5

	Owusu et al., 2018 d [25]	Cardiac Clinic	Cross-sectional study	Ashanti	Arrhythmia	432	27	6.3

	Owusu et al., 2018 e [25]	Cardiac Clinic	Cross-sectional study	Ashanti	Coronary artery disease	432	18	4.2

	Owusu et al., 2018 f [25]	Cardiac Clinic	Cross-sectional study	Ashanti	Congenital heart disease	432	15	3.5

	Owusu et al., 2018 g [25]	Cardiac Clinic	Cross-sectional study	Ashanti	Venous-thromboembolism	432	11	2.5

	Owusu et al., 2018 h [25]	Cardiac Clinic	Cross-sectional study	Ashanti	Pulmonary hypertension	432	8	1.9

	Owusu et al., 2018 i [25]	Cardiac Clinic	Cross-sectional study	Ashanti	Infective endocarditis	432	4	0.9

	Owusu et al., 2018 j [25]	Cardiac Clinic	Cross-sectional study	Ashanti	Sickle cell heart disease	432	4	0.9

	Owusu et al., 2018 k [25]	Cardiac Clinic	Cross-sectional study	Ashanti	Thyroid heart disease	432	3	0.7

	Owusu et al., 2018 m [25]	Cardiac Clinic	Cross-sectional study	Ashanti	Pericardial disorders	432	3	0.7

	Owusu et al., 2018 n [25]	Cardiac Clinic	Cross-sectional study	Ashanti	Heart failure	432	3	0.7

3	Sarfo et al., 2016 [26]	Neurology Clinic	Cross-sectional study	Ashanti	Stroke	1812	1048	57.1

4	Hayfron-Benjamin et al., 2019 [27]	Community urban	Cross-sectional study	More than one	Peripheral artery disease	1419	126	8.93

	Hayfron-Benjamin et al., 2019 [27]	Community Rural	Cross-sectional study	More than one	Peripheral artery disease	1017	76	7.52

5	Wiredu et al., 2001 [18]	Stroke Autopsy	Cross-sectional study	Greater Accra	Stroke	9760	1086	11.1

6	Amoah, 2000 a [33]	Cardiac Clinic	Cross-sectional study	Greater Accra	Hypertensive heart disease	708	133	18.8

	Amoah, 2000 b [33]	Cardiac Clinic	Cross-sectional study	Greater Accra	Rheumatic heart disease	708	123	17.4

	Amoah, 2000 c [33]	Cardiac Clinic	Cross-sectional study	Greater Accra	Cardiomyopathies	708	103	14.6

	Amoah, 2000 d [33]	Cardiac Clinic	Cross-sectional study	Greater Accra	Congenital heart disease	708	90	12.7

	Amoah, 2000 e [33]	Cardiac Clinic	Cross-sectional study	Greater Accra	Coronary artery disease	708	80	11.3

	Amoah, 2000 f [33]	Cardiac Clinic	Cross-sectional study	Greater Accra	Pericardial disorders	708	56	7.9

	Amoah, 2000 g [33]	Cardiac Clinic	Cross-sectional study	Greater Accra	Infective endocarditis	708	32	4.5

	Amoah, 2000 h [33]	Cardiac Clinic	Cross-sectional study	Greater Accra	Arrhythmia	708	13	1.8

	Amoah, 2000 i [33]	Cardiac Clinic	Cross-sectional study	Greater Accra	Pulmonary hypertension	708	12	1.7

	Amoah, 2000 j [33]	Cardiac Clinic	Cross-sectional study	Greater Accra	Thyroid heart disease	708	10	1.4

	Amoah, 2000 k [33]	Cardiac Clinic	Cross-sectional study	Greater Accra	Aortic aneurysm	708	8	1.1

	Amoah, 2000 m [33]	Cardiac Clinic	Cross-sectional study	Greater Accra	Pulmonary embolism	708	6	0.9

	Amoah, 2000 n [33]	Cardiac Clinic	Cross-sectional study	Greater Accra	Thromboembolic pulmonary hypertension	708	5	0.7

7	Edingion, 1954 [19]	General Autopsy	Cross-sectional study	Greater Accra	CVD	3645	467	12.8

8	Agongo et al., 2022 [28]	Community Rural	Cross-sectional study	Northern	CVD	1839	29	1.6

9	Amoah et al., 2000 a [16]	Cardiac Clinic	Cross-sectional study	Greater Accra	Hypertensive heart disease	572	122	21.3

	Amoah et al., 2000 b [16]	Cardiac Clinic	Cross-sectional study	Greater Accra	Rheumatic heart disease	572	115	20.1

	Amoah et al., 2000 c [16]	Cardiac Clinic	Cross-sectional study	Greater Accra	Idiopathic cardiomyopathy	572	96	16.8

	Amoah et al., 2000 d [16]	Cardiac Clinic	Cross-sectional study	Greater Accra	Congenital heart disease	572	57	10

	Amoah et al., 2000 e [16]	Cardiac Clinic	Cross-sectional study	Greater Accra	Coronary artery disease	572	56	9.8

	Amoah et al., 2000 f [16]	Cardiac Clinic	Cross-sectional study	Greater Accra	Pericardial disorders	572	44	7.7

	Amoah et al., 2000 g [16]	Cardiac Clinic	Cross-sectional study	Greater Accra	Infective endocarditis	572	25	4.4

	Amoah et al., 2000 h [16]	Cardiac Clinic	Cross-sectional study	Greater Accra	Pulmonary hypertension	572	11	1.9

	Amoah et al., 2000 i [16]	Cardiac Clinic	Cross-sectional study	Greater Accra	Thyroid heart disease	572	10	1.8

	Amoah et al., 2000 j [16]	Cardiac Clinic	Cross-sectional study	Greater Accra	Arrhythmia	572	6	1

	Amoah et al., 2000 k [16]	Cardiac Clinic	Cross-sectional study	Greater Accra	Aortic aneurysm	572	6	1

	Amoah et al., 2000 m [16]	Cardiac Clinic	Cross-sectional study	Greater Accra	Thromboembolic pulmonary hypertension	572	4	0.7

	Amoah et al., 2000 n [16]	Cardiac Clinic	Cross-sectional study	Greater Accra	Pulmonary embolism	572	4	0.7

	Amoah et al., 2000 o [16]	Cardiac Clinic	Cross-sectional study	Greater Accra	Amyloid heart disease	572	3	0.5

10	Sanuade et al., 2019 [29]	Community Urban	Cross-sectional study	More than one	Stroke	4279	112	2.6

11	Sarfo et al., 2021 [30]	HIV Clinic	Prospective cohort study	Ashanti	CVD	255	5	1.96

12	Haddock et al., 1970 a [34]	Medical admission	Cross-sectional study	Greater Accra	Heart failure	5545	539	9.7

	Haddock et al., 1970 b [34]	Medical admission	Cross-sectional study	Greater Accra	Stroke	5545	350	6.3

13	Sanuade et al., 2014 [20]	General autopsy	Cross-sectional study	Greater Accra	CVD	19289	4287	22.2

14	Sarfo et al., 2017 [17]	Neurology Clinic	Cross-sectional study	Ashanti	Stroke	934	607	65

15	Sarfo et al., 2015 [31]	General Admission	Cross-sectional study	Ashanti	Stroke	2000	19	1

	Sarfo et al., 2015 a [31]	General Admission	Cross-sectional study	Ashanti	Stroke	1132	569	50.3

	Sarfo et al., 2015 b [31]	General Admission	Cross-sectional study	Ashanti	Stroke	1132	382	33.7

	Sarfo et al., 2015 c [31]	General Admission	Cross-sectional study	Ashanti	Stroke	1132	181	16

16	Agyemang et al., 2012 a [32]	Medical Admission	Cross-sectional study	Ashanti	Stroke	1054	96	9.1

	Agyemang et al., 2012 b [32]	Medical Admission	Cross-sectional study	Ashanti	Stroke	1054	139	13.2


### 2.4 Quality assessment of the included studies

The methodological quality of the included studies was assessed independently by two authors (LST and AD) using a tool developed by Hoy et al. [22] to assess the risk of bias in prevalence studies. The risk of bias was assessed in nine categories that ranged from 0 to 9, and each item was assigned a score of 1 (yes) or 0 (no). The risk was classified as low (≥ 7), moderate (4–6), or high (≤ 3) [6,23] (Supplementary file, Table 2, on pages 2–3).

### 2.5 Statistical analysis

Data were analysed using STATA version 17. Heterogeneity across studies was assessed using the I^2^ statistic and the corresponding p-value. Heterogeneity was considered low (I^2^ = 0–25%), moderate (I^2^ = 26–50%), or high (I^2^ > 50%). Depending on the heterogeneity of the data, random-effect (for I^2^ ≥ 50%) or fixed-effect (for I^2^ < 50%) models were used. The effect sizes and pooled odds ratio (POR) were determined using the random-effects DerSimonian-Laird (DL) model. A funnel plot was used visually in conjunction with meta-regression analysis to investigate publication bias. Statistically, Egger’s regression-based and Begg’s rank correlation tests (p < 0.05) were applied to confirm publication bias. A leave-one-out sensitivity meta-analysis was performed to assess the robustness of the findings and how our pooled estimates were driven by a single study. Subgroup analyses were conducted to identify potential sources of heterogeneity in the prevalence estimates.

## 3 Results

Figure 1 presents the PRISMA flow diagram of the study search and selection procedures. The search yielded a total of 2935 study titles from the databases and reference list. After duplicate removal, 2894 study titles remained; 2837 were excluded by title and abstract screening. Of those, 57 full-text studies were reviewed, and 16 met all the inclusion criteria used in the meta-analysis because they included the necessary data to calculate the PP.

### 3.1 Assessment of the risk of bias in the included studies

Regarding the methodological quality of the included studies, 10 studies (62.5%) had a low risk of bias [17,24,25,26,27,28,29,30,31,32], and six studies (37.5%) had a moderate risk of bias [16,18,19,20,33,34]. The mean (standard deviation) risk of bias was 6.94 (±1.69) (Supplementary file, Table 2, on pages 2–3).

### 3.2 Diagnosis of CVD

CVD diagnosis was done using the World Health Organization’s (WHO) definition [5], the International Classification of Diseases, 10^th^ Revision, Clinical Modification (ICD-10-CM) Official Guidelines for Coding and Reporting (UPDATED 2023) [35] and standardised evidence-based World Heart Federation (WHF) Roadmap for Heart Failure [36]. We found one study reported amyloid heart disease, two reported aortic aneurysms, three reported arrhythmia, four reported general CVD, two reported cardiomyopathy, three reported congenital heart diseases, three reported coronary artery diseases, two reported heart failure, three reported hypertensive heart diseases, one reported idiopathic cardiomyopathy, three reported infective endocarditis, three reported pericardial disorders, two reported peripheral artery disease, two reported pulmonary embolism, three reported pulmonary hypertension, two reported rheumatic heart disease, one reported sickle cell heart disease, ten reported stroke, two reported thromboembolic pulmonary hypertension, three reported thyroid heart disease, one reported valvular heart disease and one reported venous thromboembolism (Table 1).

### 3.3 Characteristics of the included studies

The 16 included studies present data on 58912 participants between 1954 and 2022 [16,17,29,30,31,32,33,34,18,19,20,24,25,26,27,28]. Six studies each included in the meta-analysis were from the Greater Accra region [16,18,19,20,33,34] and in the Ashanti region [17,25,26,30,31,32], three studies were conducted in more than one region [24,27,29], and one was from the Northern region [28]. Fourteen of the studies were cross-sectional [16,18,31,32,33,34,19,20,24,25,26,27,28,29], and two were prospective cohort studies [24,30]. Twelve studies were hospital-based (one in the hypertension and diabetes clinic [24], three at the cardiac clinic [16,25,33], two in the neurology clinic [17,26], one in the HIV clinic [30], two from medical admission [32,34], one from general admission [31], three by autopsy reports [18,19,20], and three were community-based [27,28,29] (Table 1).

### 3.4 Meta-analysis

**Figure 2:**
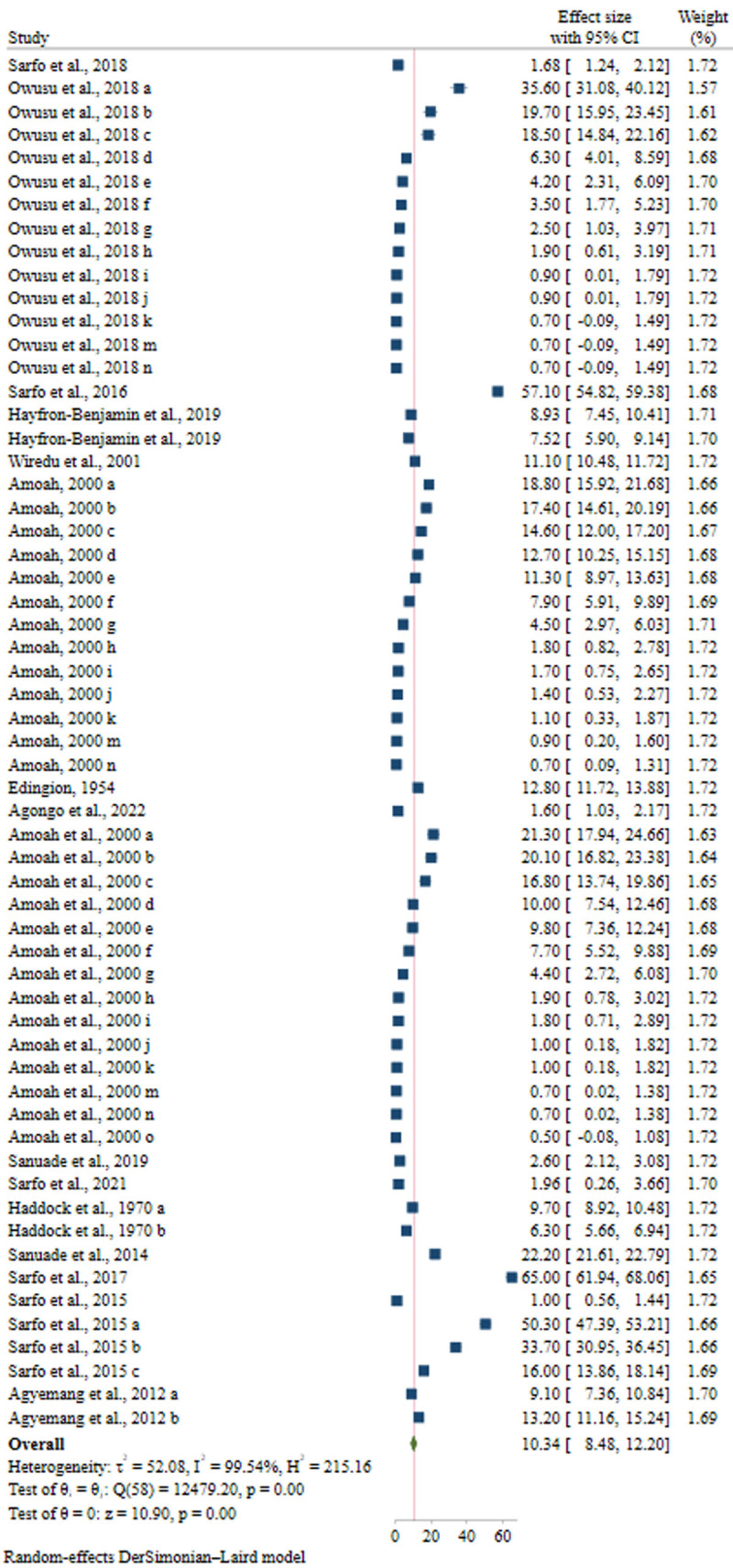
Forest plot for the prevalence of CVD in Ghana.

Sixteen studies were included in the meta-analysis, with six studies reporting more than one prevalence of CVD [16,25,31,32,33,34], giving a total of 59 estimates for PP. The PP from the random-effects DerSimonian-Laird (DL) model in the general population in Ghana was 10.34% (95% Cl: 8.48, 12.20). Heterogeneity between the studies was high and significant (l^2^ 99.54%), *p* < 0.001) (Figure 2).

### 3.5 Publication bias

Visually, the funnel plot was asymmetrical, suggesting an overall publication bias for the studies included in the meta-analyses (Supplementary file, Figure 1, on page 4). This was confirmed statistically by Egger’s regression-based test (p < 0.001) and Begg’s rank correlation test (p < 0.001).

### 3.6 Meta-regression

Meta-regression analyses were performed using variables such as year of publication, total sample size and a summary item on the overall risk of study bias to identify potential sources of heterogeneity. In bivariate analysis, only the total sample size and the summary item on the overall risk of study bias showed a significant source of heterogeneity. In the multivariable analysis, all the included variables showed a significant source of heterogeneity (Supplementary file, Table 3, on page 5).

### 3.7 Subgroup analysis

Subgroup analyses were performed based on the study’s population, study setting, study design, region and diagnosis. Significant heterogeneity was observed in the prevalence estimates of CVD across the subgroup analyses. Concerning the study setting, the highest prevalence of CVD was reported among patients who visited the neurologic clinic 60.98% (95% CI: 53.24, 68.72), followed by patients who came for general admission 25.22% (95% CI: 3.23, 47.21). The prevalence of CVD was 10.65% (95% Cl: 8.69, 12.62) in studies conducted using a cross-sectional design. The prevalence of CVD was higher in hospital-based settings 10.74% (95%, Cl: 8.69, 12.79) than in community-based settings 5.04% (95% Cl: 2.54, 7.53). The PP of CVD was 15.48% (95% Cl: 11.01, 19.94) and 7.86% (95% Cl: 5.47, 10.25) in the Ashanti Region and Greater Accra Region, respectively. We also performed a subgroup meta-analysis based on the CVD diagnosis. Hypertensive heart disease had the highest PP of 25.08% (95% CI: 16.11, 34.05), followed by stroke at 24.08% (95% CI: 16.62, 27.53%) and valvular heart disease at 19.70% (95% CI: 15.95, 23.45%) (Table 2).

**Table 2:** Subgroup analysis regarding the prevalence of CVD in Ghana.


SUBGROUPS	POOLED PREVALENCE 95% CONFIDENCE INTERVAL (CI) (%)	HETEROGENEITY ACROSS THE STUDIES		HETEROGENEITY BETWEEN GROUPS (p-VALUE)

		I^2^ (%)	p-VALUE	

**Total**	**10.34[8.48, 12.20]**	**99.54**	**<0.001**	**<0.001**

**Study setting**				

Cardiac Clinic	6.55[5.45, 7.66]	97.41	<0.001	<0.001

Community Rural	4.51[–1.29-10.31]	97.80	<0.001	

Community Urban	5.72[–0.48, 11.93]	98.42	<0.001	

General Admission	25.22[3.23, 47.21]	99.82	<0.001	

General Autopsy	17.51[8.3, 26.72]	99.55	<0.001	

HIV Clinic	1.96[0.26, 3.66]			

Hypertension and Diabetes Clinic	1.68[1.24, 2.12]			

Medical Admission	9.46[6.87, 12.05]	95.82	<0.001	

Neurology Clinic	60.98[53.24, 68.72]	93.93	<0.001	

Stroke Autopsy	11.10[10.48, 11.72]			

**Study setting classification**				

Community-based	5.04[2.54, 7.53]	97.41	<0.001	0.001

Hospital-based	10.74[8.69, 12.79]	99.56	<0.001	

**Study design**				

Cross-sectional study	10.65[8.69, 12.62]	99.55	<0.001	<0.001

Prospective cohort study	1.70[1.27, 2.13]	0.00	0.755	

**Region**				

Ashanti Region	15.48[11.01, 19.94]	99.65	<0.001	<0.001

Greater Accra Region	7.86[5.47, 10.25]	99.48	<0.001	

More than one region	5.03[2.72, 7.34]	97.54	<0.001	

Northern Region	1.60[1.03, 2.17]			

**Diagnosis**				

Amyloid heart disease	0.50[–0.08, 1.08]			<0.001

Aortic aneurysm	1.05[0.49, 1.61]	0.00	0.861	

Arrhythmia	2.70[0.63, 4.77]	89.1	<0.001	

General CVD	9.65[–2.20, 21.49]	99.88	<0.001	

Cardiomyopathies	16.33[12.53, 20.13]	65.48	<0.001	

Congenital heart disease	8.68[2.89, 14.47]	95.20	<0.001	

Coronary artery disease	8.39[3.86, 12.91]	92.05	<0.001	

Heart failure	5.20[–3.62, 14.02]	99.61	<0.001	

Hypertensive heart disease	25.08[16.11, 34.05]	94.88	<0.001	

Idiopathic cardiomyopathy	16.80[13.74, 19.86]			

Infective endocarditis	3.20[0.54, 5.86]	91.47	<0.001	

Pericardial disorders	5.37[–0.14, 10.88]	97.14	<0.001	

Peripheral artery disease	8.26[6.86, 9.64]	36.78	0.209	

Pulmonary embolism	0.80[0.31, 1.29]	0.00	0.688	

Pulmonary hypertension	1.81[1.18, 2.44]	0.00	0.954	

Rheumatic heart disease	18.61[15.98, 21.23]	33.64	0.220	

Sickle cell heart disease	0.90[0.01, 1.79]			

Stroke	24.08[16.62, 27.53]	99.82	<0.001	

Thromboembolic pulmonary hypertension	0.70[0.24, 1.16]	0.00	1.000	

Thyroid heart disease	1.22[0.60, 1.85]	31.57	0.232	

Valvular heart disease	19.70[15.95, 23.45]			

Veno-thromboembolism	2.50[1.03, 3.97]			


### 3.8 Sensitivity analysis

A sensitivity analysis was performed using a random-effects model, and the results showed that no single study affected the PP of CVD. After a single study was removed from the meta-analysis, the pooled prevalence was close to the actual effect size, which implies the absence of a single study effect on an overall study (Supplementary file, Table 4, on pages 6–8).

### 3.9 Risk factors for CVD

Eight out of 16 included studies reported the risk factors for CVD [17,18,24,28,29,30,31,32] (Table 3).

**Table 3:** Risk factors of CVD in Ghana.


NUMBER	RISK FACTORS	STUDY	OR (95% CI)

1	Male gender	Sarfo et al., 2018 [24]	2.10 [1.21, 3.64]

		Wiredu et al., 2001 [18]	2.07 [1.75, 2.45]

		Agyemang et al., 2012 [32]	1.14 [0.98, 1.27]

		**Overall, DL (I**^2^ **= 93.8%, p < 0.001)**	**1.66 [1.02, 2.70]**

2	Old age	Sarfo et al., 2018 [24]	1.28 [1.03, 1.60]

		Sarfo et al., 2015 [31]	1.31 [1.16, 1.47]

		Sarfo et al., 2017 [17]	1.41 [1.15, 1.73]

		**Overall, DL (1**^2^ **= 0.0%, p = 0.784)**	**1.32 [1.21, 1.45]**

3	Unemployment	Sarfo et al., 2018 [24]	1.89 [1.11, 3.23]

		Sanuade et al., 2019 [29]	3.78 [2.02, 7.07]

		**Overall, DL (I**^2^ **= 63.3%, p = 0.099)**	**2.62 [1.33, 5.16]**

4	Diabetes	Sanuade et al., 2019 [29]	3.95 [1.88, 8.30]

		Sarfo et al., 2017 [17]	2.24 [1.32, 3.80]

		**Overall, DL (I**^2^ **= 32.8%, p = 0.223)**	**2.79 [1.62, 4.81]**

5	Hypertension	Sanuade et al., 2019 [29]	3.01 [1.77, 5.13]

		Sarfo et al., 2021 [30]	8.61 [1.32, 56.04]

		**Overall, DL (I**^2^ **= 10.5%, p = 0.290)**	**3.41 [1.75, 6.66]**

6	Smoking	Sarfo et al., 2018 [24]	2.59 [1.18, 5.67]

7	Physical inactivity	Sarfo et al., 2018 [24]	1.81 [1.06, 3.10]

8	Divorced	Sanuade et al., 2019 [29]	2.47 [1.22, 4.97]

9	Rise in CD4 count	Sarfo et al., 2021 [30]	0.56 [0.35, 0.88]

10	Non-HDL-C	Agongo et al., 2022 [28]	1.58 [1.05, 2.39]

11	LDL-C/HDL-C levels	Agongo et al., 2022 [28]	1.26 [1.00, 1.59]


OR: odds ratio; *CD4:* clusters of differentiation 4; HDL-C: high-density lipoprotein cholesterol; TC: total cholesterol; LDL-C: low-density lipoprotein cholesterol; DL: random-effects DerSimonian-Laird model.

#### 3.9.1 Male gender

Three studies including 14034 participants diagnosed with stroke reported an association between male gender and CVD [18,24,32]. The POR showed that males were 1.66 times more likely to develop CVD (POR: 1.66; 95% Cl: 1.02, 2.70, I^2^ = 93.8%, p < 0.001) than females (Table 3).

#### 3.9.2 Old age

A total of three studies involving 6154 participants diagnosed with stroke reported the association between increasing age and CVD [17,24,31]. Older participants were 1.32 times more likely to develop CVD (POR: 1.32; 95% CI: 1.21, 1.45; I^2^ = 0.0%; p = 0.784) than those who were younger (Table 3).

#### 3.9.3 Unemployment

Two studies comprising 7499 participants diagnosed with stroke reported an association between unemployment and CVD [24,29]. The odds of developing CVD among the unemployed participants were 2.62 times (POR: 2.62; 95% Cl: 1.33, 5.16, I^2^ = 63.3%, p = 0.099) the odds among those who were employed (Table 3).

#### 3.9.4 Diabetes

Two studies including 5213 participants diagnosed with stroke reported the association between diabetes and CVD [17,29]. The POR showed that those with diabetes were 2.79 times more likely to have reported CVD (POR: 2.79; 95% Cl: 1.62, 4.81, I^2^ = 32.8%, p = 0.223) than those without diabetes (Table 3).

#### 3.9.5 Hypertension

A total of two studies comprising 4534 participants diagnosed with stroke (n=4279) and general CVD (n=255) reported the association between hypertension and CVD [29,30]. The POR revealed that those with hypertension were 3.41 times more likely to develop CVD (POR: 3.41; 95% Cl: 1.75, 6.66, I^2^ = 10.5%, p = 0.290) than those without hypertension (Table 3).

#### 3.9.6 Other risk factors

Only one study reported smoking (odds ratio [OR] = 2.59; 95% Cl: 1.18, 5.67) and physical inactivity (OR: 1.81; 95% Cl: 1.06, 3.10) [24], divorce (OR: 2.47; 95% Cl: 1.22, 4.97) [29], a rise in CD4 count (OR: 0.56; 95% Cl: 0.35, 0.88) [30], non-HDL-C levels (OR: 1.58; 95% Cl: 1.05, 2.39) and LDL-C/HDL-C levels (OR: 1.26; 95% Cl: 1.00, 1.59) [28] as the risk factors of CVD (Table 3).

## 4 Discussion

The prevalence of CVD is particularly difficult to estimate in a population because it is a group of disorders of the heart and blood vessels rather than a single disease. The current systematic review and meta-analysis examined the pooled prevalence (PP) of CVD and risk factors in Ghana. Our meta-analysis showed that the PP of CVD was 10.34%, which was higher than the prevalence of 5.5% in SSA [10], 10.1% in the Middle East [4], the global prevalence of 4.2% from 1990 to 2015 [9] and 9.1% in the United States of America [37]. Our PP was also higher than the prevalence of 5% in a meta-analysis conducted in Ethiopia [6]. The high PP of CVD in Ghana is not surprising, given that the bulk of data available was from institutional settings (e.g., cardiac and stroke clinics). However, the PP of CVD in the current study was lower than what was reported in some countries from community-based studies: 24.8% in India [38], 19.3% in China [39], 13.3% in Gabon [40], 26.1% in Lebanon [13], 23% in Tanzania [41] and 39.2% in SSA [42]. The observed higher prevalence could be attributed to the fact that these studies are from single studies, while ours was based on the pooled estimate from several studies. There was high heterogeneity among the included studies, which was explainable by the significance of the subgroup analyses of the study setting, region in Ghana, diagnostic criteria of CVD and the presence of publication bias. The highest risk factors of CVD identified were hypertension and diabetes, which was confirmed by Abban et al. [8] and Keates et al. [2] in earlier studies in Africa.

In this review, the data showed that there was a rise in CVD prevalence from a study conducted in 2016, and it dropped in a study conducted in 2018. A similar finding was reported in a study conducted in Ethiopia, where there was a high increase in CVD prevalence from 2008 to 2013, and it declined in a 2015 study [6]. However, the prevalence of CVD from a systematic analysis of data in SSA showed no decline in CVD from 1990 to 2013 [10], as well as in the Chinese population from 2005 to 2020 [10] and the global prevalence from 1990 to 2019 [3]. These disparities across the studies could be attributed to improved medical care and public health interventions as well as upgraded medical technology for proper diagnosis and effective treatment of CVD and CVD risk factors.

In our review, we identified stroke as the most frequently reported CVD, followed by generally or aggregated reported CVD (see Table 3). However, in our subgroup meta-analysis of CVD diagnosis, hypertensive heart disease had the highest PP of 25.1%, followed by stroke (24.3%) and valvular heart disease (19.7%) (Table 2). In contrast, studies conducted in India [38] and China [39] and systematic analysis of data from SSA [10] have reported ischemic heart disease as the CVD with the highest prevalence. Similar to our findings, another systematic review and meta-analysis conducted in SSA [42] reported hypertensive heart disease as the CVD with the highest prevalence. Globally [3] and in several SSA countries [2], ischemic heart disease and stroke have been identified as the topmost CVD and result in 32% of all global mortality [3]. However, the diagnosis of ischaemic heart diseases in SSA is primarily based on electrocardiogram or echocardiogram findings, which have high sensitivity but low specificity compared to the use of advanced imaging techniques (coronary angiogram, radionuclear studies and cardiac magnetic resonance imaging) in other regions of the world. Hence, the diagnosis of ischaemic heart diseases or coronary artery diseases in SSA may have some inaccuracies or misdiagnoses and subsequently overestimate the prevalence rates in SSA [43].

We also classified the prevalence of the study based on the settings (hospital-based and community-based), and the PP of CVD in hospital-based settings (10.7%) was two times higher than that in community-based settings (5.0%). Similarly, a study conducted in Ethiopia [6] showed that the PP of CVD among people admitted to hospitals was 8%, four times higher than that of the general population (2%). However, these rates were lower than the rates found in our current study. The following factors could contribute to the observed disparities. First, hospital settings provide data on patients who are already diagnosed or at risk of CVDs or are sick. Second, the diagnoses of CVD in hospital settings are more accurate than those in community settings, which can be due to a lack of diagnostic equipment. Additionally, diagnosis in the community is based on symptoms and signs.

In this systematic review, we identified risk factors associated with the high prevalence of CVD, such as male gender, old age, unemployment, diabetes and hypertension. The higher prevalence of CVD in men in our study is consistent with earlier findings in China [39], the United States of America [44] and Lebanon [13], which found an increased OR of CVD in males compared to females. Our finding, however, contradicts a study conducted using data from the global burden of disease in SSA [10], in a population-based cohort study in Italy [45] and Tanzania [41]. However, studies conducted in Ethiopia [6,7] and Gabon [40] showed no association between gender and CVD. The likely reasons for the disparities are unclear but may be due to several factors, such as different distributions of menopausal women in the studies [6,46], gender differences in health-seeking behaviour and access to CVD healthcare in Africa [47,48]. For example, premenopausal women have eight to ten years of protection from heart disease compared with men [6,46].

Older participants were more likely than young people to have CVD, which is consistent with a study conducted in Tanzania [41], Pakistan [49], Somalia [1], Lebanon [13] and Ethiopia [7]. The most likely explanation is that ageing has a degenerative effect on blood vessels, leading to increased atherosclerotic CVD, such as myocardial infarction and infarction stroke [7,50]. Inconsistent with studies conducted in populations in Tanzania [41] and Ethiopia [7] that found no association between unemployment and CVD, the likelihood of the association between the unemployed and CVD was approximately three times that of the employed in our current study in Ghana. The likely reason for this is that unemployment leads to psychosocial stress and subsequently CVD [51].

Persons with diabetes mostly experience insulin resistance, which is associated with increased cardiovascular risk [52]. In our meta-analysis, the participants with diabetes were more likely to develop CVD than those without diabetes. This finding is consistent with findings reported in earlier systematic reviews and meta-analyses [4,53] and studies conducted in Somalia [1], Lebanon [13], the United States of America [44] and Ethiopia [54]. The likely explanation is that hyperglycemia plays a central role in the pathogenesis of vascular diseases, as evidenced by the increased prevalence of atherosclerosis in people with diabetes without dyslipidemia or hypertension [55]. Hypertension causes pressure loading on the heart (causing hypertrophy, diastolic and systolic dysfunction), arrhythmias (e.g., atrial fibrillation), coronary artery diseases and aorta/aortic valve diseases; all these effects constitute hypertensive heart disease [8,56]. This review has shown an increased likelihood of having CVD in hypertensive patients compared to those without hypertension. Similar findings were reported in systematic reviews and meta-analyses [4,53] and studies conducted in Lebanon [13], Gabon [40], the United States of America [44], Ethiopia [7] and Tanzania [41]. Hypertension is the top killer and main cause of admissions, with 67% of all deaths in KBTH, Ghana [8].

## 5 Strengths and Limitations

The strength of this study is that it is the first meta-analysis focused on the PP of CVD and the risk factors in Ghana. The findings are beneficial to policymakers such as the Ghana Heart Initiative for health decision-making and policy guidelines for the prevention and management of CVD and its risk factors. However, some limitations need to be considered. First, the studies included in this review differed in setting, design, region, diagnosis and year of study. Second, the study also spans from 1954, diagnostic procedures have evolved, and differences in diagnostic procedures may also affect the observed prevalence of CVD. Third, we also observed significant heterogeneity between the studies; therefore, care should be taken when interpreting the PP estimates.

## 6 Conclusion and Recommendation

Our findings show a high prevalence of CVD in Ghana. The risk factors identified were male gender, older, unemployed, diabetic and hypertensive were positively associated with CVD. This study’s findings suggest the need for prevention and treatment initiatives to reduce the CVD burden in Ghana. Future studies should provide detailed descriptions of CVD, and their risk factors in Ghana, ideally from community settings, to assess the actual burden of CVD in Ghana. Furthermore, future studies should use the same diagnostic criteria used in the present study such as ICD-10-CM updated 2023 and WHF Roadmap for Heart Failure to diagnose CVD which would improve comparability.

## Data Accessibility Statement

The manuscript contains all pertinent information.

## Additional Files

The additional files for this article can be found as follows:

10.5334/gh.1307.s1 Supplementary file Table 1. The search strategy of the databases (page 1).

10.5334/gh.1307.s2 Supplementary file Table 2. Quality assessment of the included studies (pages 2–3).

10.5334/gh.1307.s3 Supplementary file Figure 1. Funnel plot for risk of publication bias for the pooled prevalence of CVD in Ghana (page 4).

10.5334/gh.1307.s4 Supplementary file Table 3. Meta-regression analysis of identified sources of heterogeneity of CVD in the current meta-analysis (page 5).

10.5334/gh.1307.s5 Supplementary file Table 4. Sensitivity analysis for a single study influences the overall study of CVD prevalence in Ghana (pages 6–8).

## Abbreviations

CVD: Cardiovascular Disease

PP: Pooled Prevalence

LMICs: Low-Middle-Income Countries

SSA: Sub-Saharan Africa

WHO: World Health Organization

POR: Pooled Odds Ratio

DL: DerSimonian-Laird model

CAD: Coronary Artery Disease

PAD: Peripheral Arterial Disease

RHD: Rheumatic Heart Disease

DVT: Deep Vein Thrombosis

CHD: Congenital Heart Disease

PE: Pulmonary Embolism
