# Supplementary material for: Prevalence of Cardiovascular Disease and Risk Factors in Ghana: A Systematic Review and Meta-analysis
Source: Glob Heart. 2024 Feb 20;19(1):21. doi: 10.5334/gh.1307 (PMC10885824; doi:10.5334/gh.1307)
Supplement: Supplementary file Table 1. — The search strategy of the databases (page 1). [file gh-19-1-1307-s1.pdf]

Table 1 Search strategy of the databases

No	Database	Search strategy	Search results
1	PubMed	("Prevalence"[MeSH Terms] OR "Prevalence"[Text Word] OR "prevalen*"[Text Word]) AND ("risk factors"[MeSH Terms] OR "risk factors"[Text Word]) AND ("Cardiovascular Diseases"[MeSH Terms] OR "Cerebrovascular Disorders"[MeSH Terms] OR "Heart Diseases"[MeSH Terms] OR "Heart Failure"[MeSH Terms] OR "Myocardial Ischemia"[MeSH Terms] OR "Coronary Artery Disease"[MeSH Terms] OR "Peripheral Arterial Disease"[MeSH Terms] OR "Rheumatic Heart Disease"[MeSH Terms] OR "Venous Thrombosis"[MeSH Terms] OR "heart defects, congenital"[MeSH Terms] OR "Pulmonary Embolism"[MeSH Terms] OR "Stroke"[MeSH Terms]) AND "Ghana"[MeSH Terms]	126
2	Google Scholar	allintitle: Ghana "prevalence" OR "cardiovascular diseases" OR "cerebrovascular disorders" OR "heart disease" OR "Pulmonary Embolism" OR "Venous Thrombosis" OR "myocardial ischemia" OR "coronary artery disease" OR "Peripheral Arterial Disease" OR "stroke" AND "risk factors"	1350
3	Cochrane Library	prevalen* AND risk factor* AND ‘cardiovascular disease* OR cerebrovascular disorder* OR heart disease* OR heart failure OR myocardial ischemia OR coronary artery disease OR peripheral arterial disease OR rheumatic heart disease OR deep vein thrombosis OR congenital heart disease OR pulmonary embolism OR stroke AND Ghana	45
4	Science Direct	Find articles with these terms: “prevalence” AND “risk factors” AND “cardiovascular diseases” AND “Ghana”	1027
5	African Journal Online	Find articles with these terms: “prevalence” AND "risk factors" AND “cardiovascular diseases” OR "coronary artery disease" AND “Ghana”	382
	Total		2930
