# Supplementary material for: Prevalence of Cardiovascular Disease and Risk Factors in Ghana: A Systematic Review and Meta-analysis
Source: Glob Heart. 2024 Feb 20;19(1):21. doi: 10.5334/gh.1307 (PMC10885824; doi:10.5334/gh.1307)
Supplement: Supplementary file Table 2. — Quality assessment of the included studies (pages 2–3). [file gh-19-1-1307-s2.pdf]

Questions for the quality assessment of the included studies

External validity

Q1. Was the study's target population a close representation of the national population?

Q2. Was the sampling frame a true or close representation of the target population?

Q3. Was some form of random selection used to select the sample OR was a census used?

Q4. Was the likelihood of nonresponse bias minimal?

Internal validity

Q5. Were data collected directly from the subjects?

Q6. Was an acceptable case definition used in the study?

Q7. Was the study instrument that measured the parameter of interest shown to have validity and reliability?

Q8. Was the same mode of data collection used for all subjects?

Q9. Was the length of the shortest prevalence period for the parameter of interest appropriate?

Q10. Were the numerator(s) and denominator(s) for the parameter of interest appropriate?

11. Summary item on the overall risk of study bias

12. Overall agreement for the 11 items

Table 2 Quality assessment of the included studies

No of the included studies	Study	External validity				Internal validity						11	12
		Q1	Q2	Q3	Q4	Q5	Q6	Q7	Q8	Q9	Q10		
1	Sarfo et al., 2018	1	1	1	0	1	1	1	1	1	Yes	8	Low
2	Owusu et al., 2018 a	1	1	0	1	0	1	0	1	1	Yes	7	Low
	Owusu et al., 2018 b	1	1	0	1	0	1	0	1	1	Yes	7	Low
	Owusu et al., 2018 c	1	1	0	1	0	1	0	1	1	Yes	7	Low
	Owusu et al., 2018 d	1	1	0	1	0	1	0	1	1	Yes	7	Low
	Owusu et al., 2018 e	1	1	0	1	0	1	0	1	1	Yes	7	Low
	Owusu et al., 2018 f	1	1	0	1	0	1	0	1	1	Yes	7	Low
	Owusu et al., 2018 g	1	1	0	1	0	1	0	1	1	Yes	7	Low
	Owusu et al., 2018 h	1	1	0	1	0	1	0	1	1	Yes	7	Low
	Owusu et al., 2018 i	1	1	0	1	0	1	0	1	1	Yes	7	Low
	Owusu et al., 2018 j	1	1	0	1	0	1	0	1	1	Yes	7	Low
	Owusu et al., 2018 k	1	1	0	1	0	1	0	1	1	Yes	7	Low
	Owusu et al., 2018 m	1	1	0	1	0	1	0	1	1	Yes	7	Low
	Owusu et al., 2018 n	1	1	0	1	0	1	0	1	1	Yes	7	Low
3	Sarfo et al., 2016	1	1	1	0	1	1	1	1	1	Yes	8	Low
4	Hayfron-Benjamin et al., 2019	1	1	0	1	1	1	1	1	1	Yes	8	Low

	Hayfron-Benjamin et al., 2019	1	1	0	1	1	1	1	1	1	Yes	8	Low
5	Wiredu et al., 2001	1	1	0	0	1	0	1	0	1	Yes	5	Moderate
6	Amoah, 2000 a	1	1	1	0	1	0	1	1	0	Yes	6	Moderate
	Amoah, 2000 b	1	1	1	0	1	0	1	1	0	Yes	6	Moderate
	Amoah, 2000 c	1	1	1	0	1	0	1	1	0	Yes	6	Moderate
	Amoah, 2000 d	1	1	1	0	1	0	1	1	0	Yes	6	Moderate
	Amoah, 2000 e	1	1	1	0	1	0	1	1	0	Yes	6	Moderate
	Amoah, 2000 f	1	1	1	0	1	0	1	1	0	Yes	6	Moderate
	Amoah, 2000 g	1	1	1	0	1	0	1	1	0	Yes	6	Moderate
	Amoah, 2000 h	1	1	1	0	1	0	1	1	0	Yes	6	Moderate
	Amoah, 2000 i	1	1	1	0	1	0	1	1	0	Yes	6	Moderate
	Amoah, 2000 j	1	1	1	0	1	0	1	1	0	Yes	6	Moderate
	Amoah, 2000 k	1	1	1	0	1	0	1	1	0	Yes	6	Moderate
	Amoah, 2000 m	1	1	1	0	1	0	1	1	0	Yes	6	Moderate
	Amoah, 2000 n	1	1	1	0	1	0	1	1	0	Yes	6	Moderate
7	Edingion, 1954	1	0	0	1	0	1	0	0	1	Yes	4	Moderate
8	Agongo et al., 2022	1	1	1	0	1	1	1	1	1	Yes	8	Low
9	Amoah et al., 2000 a	1	1	1	1	1	0	1	0	0	Yes	6	Moderate
	Amoah et al., 2000 b	1	1	1	1	1	0	1	0	0	Yes	6	Moderate
	Amoah et al., 2000 c	1	1	1	1	1	0	1	0	0	Yes	6	Moderate
	Amoah et al., 2000 d	1	1	1	1	1	0	1	0	0	Yes	6	Moderate
	Amoah et al., 2000 e	1	1	1	1	1	0	1	0	0	Yes	6	Moderate
	Amoah et al., 2000 f	1	1	1	1	1	0	1	0	0	Yes	6	Moderate
	Amoah et al., 2000 g	1	1	1	1	1	0	1	0	0	Yes	6	Moderate
	Amoah et al., 2000 h	1	1	1	1	1	0	1	0	0	Yes	6	Moderate
	Amoah et al., 2000 i	1	1	1	1	1	0	1	0	0	Yes	6	Moderate
	Amoah et al., 2000 j	1	1	1	1	1	0	1	0	0	Yes	6	Moderate
	Amoah et al., 2000 k	1	1	1	1	1	0	1	0	0	Yes	6	Moderate
	Amoah et al., 2000 m	1	1	1	1	1	0	1	0	0	Yes	6	Moderate
	Amoah et al., 2000 n	1	1	1	1	1	0	1	0	0	Yes	6	Moderate
	Amoah et al., 2000 o	1	1	1	1	1	0	1	0	0	Yes	6	Moderate
10	Sanuade et al., 2019	1	1	1	1	1	1	1	1	1	Yes	9	Low
11	Sarfo et al., 2021	1	1	1	1	1	1	1	0	1	Yes	8	Low
12	Haddock et al., 1970 a	1	0	0	0	1	1	0	0	1	Yes	4	Moderate
	Haddock et al., 1970 b	1	0	0	0	1	1	0	0	1	Yes	4	Moderate
13	Sanuade et al., 2014	1	0	1	0	1	0	1	0	1	Yes	5	Moderate
14	Sarfo et al., 2017	1	1	1	0	1	1	1	1	1	Yes	8	Low
15	Sarfo et al., 2015	1	1	1	1	1	1	0	1	1	Yes	8	Low
	Sarfo et al., 2015 a	1	1	1	1	1	1	0	1	1	Yes	8	Low
	Sarfo et al., 2015 b	1	1	1	1	1	1	0	1	1	Yes	8	Low
	Sarfo et al., 2015 c	1	1	1	1	1	1	0	1	1	Yes	8	Low
16	Agyemang et al., 2012 a	1	1	1	1	1	1	1	1	1	Yes	9	Low
	Agyemang et al., 2012 b	1	1	1	1	1	1	1	1	1	Yes	9	Low
