# Supplementary figures and images for: Prevalence of Cardiovascular Disease and Risk Factors in Ghana: A Systematic Review and Meta-analysis
Source: Glob Heart. 2024 Feb 20;19(1):21. doi: 10.5334/gh.1307 (PMC10885824; doi:10.5334/gh.1307)

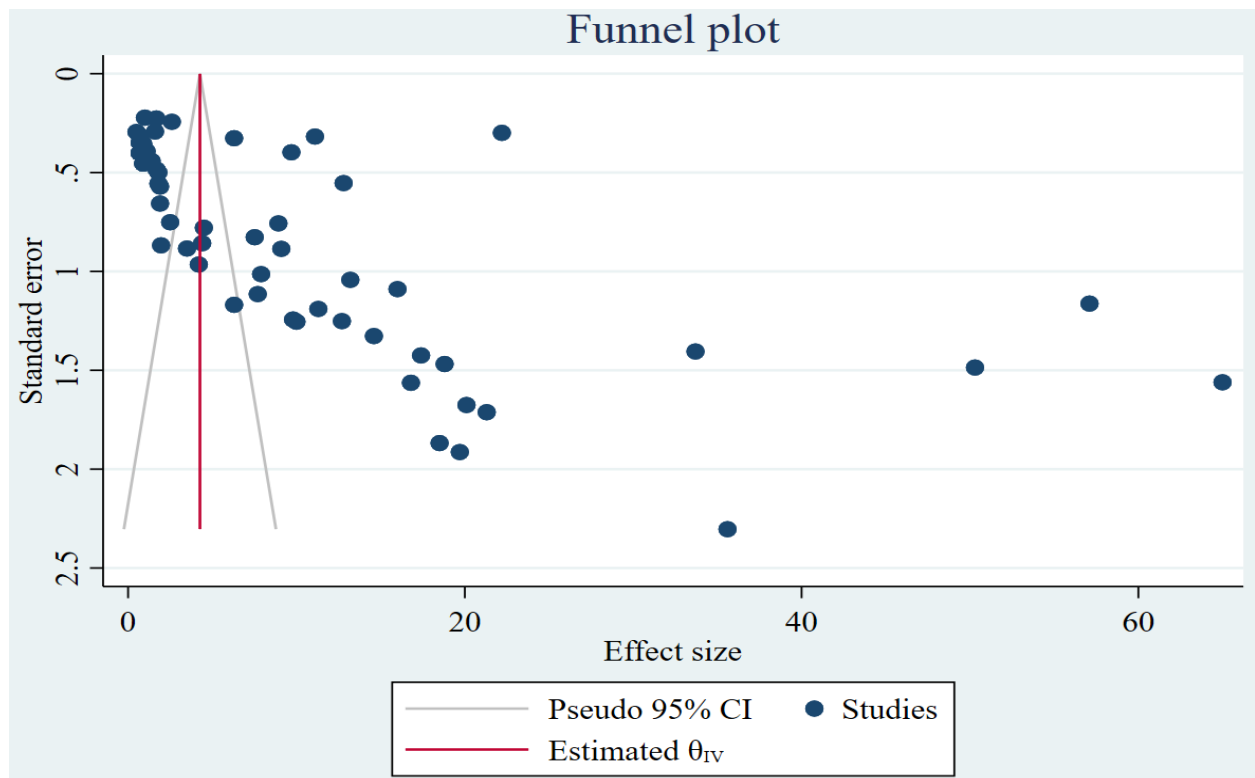

**Figure 1: Funnel plot for risk of publication bias for the pooled prevalence of CVD in Ghana**

Supplement: Supplementary file Figure 1. — Funnel plot for risk of publication bias for the pooled prevalence of CVD in Ghana (page 4). [file gh-19-1-1307-s3.pdf]
