# Supplementary material for: Prevalence of Cardiovascular Disease and Risk Factors in Ghana: A Systematic Review and Meta-analysis
Source: Glob Heart. 2024 Feb 20;19(1):21. doi: 10.5334/gh.1307 (PMC10885824; doi:10.5334/gh.1307)
Supplement: Supplementary file Table 3. — Meta-regression analysis of identified sources of heterogeneity of CVD in the current meta-analysis (page 5). [file gh-19-1-1307-s4.pdf]

Table 3 Meta-regression analysis of identified sources of heterogeneity of CVD in the current meta-analysis

Variable	Bivariate		Multivariate	
	Coefficient	(95% CI), p-value	Coefficient	(95% CI), p-value
Year of publication	0.122	(-0.022, 0.266), 0.098	-0.231	(-0.446, -0.016), 0.035
Total sample size	0.001	(0.000, 0.001), 0.046	0.001	(0.000, 0.002), 0.001
The summary item on the overall risk of study bias	2.426	(0.811, 4.041), 0.003	5.335	(18.944, 854.541), <0.001
