# Supplementary material for: Prevalence of Cardiovascular Disease and Risk Factors in Ghana: A Systematic Review and Meta-analysis
Source: Glob Heart. 2024 Feb 20;19(1):21. doi: 10.5334/gh.1307 (PMC10885824; doi:10.5334/gh.1307)
Supplement: Supplementary file Table 4. — Sensitivity analysis for a single study influences the overall study of CVD prevalence in Ghana (pages 6–8). [file gh-19-1-1307-s5.pdf]

Table 4 Sensitivity analysis for single study influence on the overall study of CVD prevalence in Ghana

No of study	Author (publication year)	Sample size	Estimation	Lower limit	Upper limit
1	Sarfo et al., 2018	3220	10.50	8.56	12.44
2	Owusu et al., 2018 a	432	9.93	8.07	11.79
	Owusu et al., 2018 b	432	10.18	8.31	12.05
	Owusu et al., 2018 c	432	10.20	8.33	12.07
	Owusu et al., 2018 d	432	10.41	8.53	12.29
	Owusu et al., 2018 e	432	10.45	8.57	12.32
	Owusu et al., 2018 f	432	10.46	8.58	12.34
	Owusu et al., 2018 g	432	10.48	8.59	12.36
	Owusu et al., 2018 h	432	10.49	8.60	12.37
	Owusu et al., 2018 i	432	10.51	8.62	12.40
	Owusu et al., 2018 j	432	10.51	8.62	12.40
	Owusu et al., 2018 k	432	10.51	8.62	12.40
	Owusu et al., 2018 m	432	10.51	8.62	12.40
	Owusu et al., 2018 n	432	10.51	8.62	12.40
3	Sarfo et al., 2016	1812	9.51	7.79	11.22
4	Hayfron-Benjamin et al., 2019	1419	10.36	8.49	12.24
	Hayfron-Benjamin et al., 2019	1017	10.39	8.51	12.27
5	Wiredu et al., 2001	9760	10.33	8.45	12.20
6	Amoah, 2000 a	708	10.20	8.33	12.06
	Amoah, 2000 b	708	10.22	8.35	12.09
	Amoah, 2000 c	708	10.27	8.39	12.14
	Amoah, 2000 d	708	10.30	8.42	12.17
	Amoah, 2000 e	708	10.32	8.45	12.20
	Amoah, 2000 f	708	10.38	8.50	12.26
	Amoah, 2000 g	708	10.44	8.56	12.32

	Amoah, 2000 h	708	10.49	8.60	12.38
	Amoah, 2000 i	708	10.49	8.60	12.38
	Amoah, 2000 j	708	10.50	8.61	12.39
	Amoah, 2000 k	708	10.50	8.61	12.40
	Amoah, 2000 m	708	10.51	8.61	12.41
	Amoah, 2000 n	708	10.51	8.61	12.42
7	Edingion, 1954	3645	10.29	8.43	12.16
8	Agongo et al., 2022	1839	10.50	8.58	12.41
9	Amoah et al., 2000 a	572	10.16	8.29	12.02
	Amoah et al., 2000 b	572	10.18	8.31	12.04
	Amoah et al., 2000 c	572	10.23	8.36	12.10
	Amoah et al., 2000 d	572	10.34	8.47	12.22
	Amoah et al., 2000 e	572	10.35	8.47	12.22
	Amoah et al., 2000 f	572	10.38	8.51	12.26
	Amoah et al., 2000 g	572	10.44	8.56	12.32
	Amoah et al., 2000 h	572	10.49	8.60	12.37
	Amoah et al., 2000 i	572	10.49	8.60	12.38
	Amoah et al., 2000 j	572	10.51	8.61	12.40
	Amoah et al., 2000 k	572	10.51	8.61	12.40
	Amoah et al., 2000 m	572	10.51	8.61	12.41
	Amoah et al., 2000 n	572	10.51	8.61	12.41
	Amoah et al., 2000 o	572	10.52	8.61	12.42
10	Sanuade et al., 2019	4279	10.49	8.55	12.42
11	Sarfo et al., 2021	255	10.48	8.61	12.36
12	Haddock et al., 1970 a	5545	10.35	8.47	12.24
	Haddock et al., 1970 b	5545	10.42	8.51	12.32
13	Sanuade et al., 2014	19289	10.07	8.46	11.68
14	Sarfo et al., 2017	934	9.40	7.64	11.16
15	Sarfo et al., 2015	2000	10.51	8.58	12.45

	Sarfo et al., 2015 a	1132	9.65	7.85	11.46
	Sarfo et al., 2015 b	1132	9.94	8.09	11.78
	Sarfo et al., 2015 c	1132	10.24	8.37	12.11
16	Agyemang et al., 2012 a	1054	10.36	8.48	12.24
	Agyemang et al., 2012 b	1054	10.29	8.42	12.16
Combined			10.34	8.48	12.20
